# Accelerating progress towards better public health and sustainable development in fragile settings

**DOI:** 10.1136/bmjgh-2025-020653

**Published:** 2025-11-19

**Authors:** Rawlance Ndejjo, Daniel Helldén, Irene Wanyana, Rage Adem, Landry Egbende, Hassan W Nor, Branly Mbunga, Mattias Schedwin, Mala Ali Mapatano, Tobias Alfvén, Rhoda K. Wanyenze, Nina Viberg

**Affiliations:** 1 Department of Disease Control and Environmental Health, School of Public Health, Makerere University, Kampala, Uganda; 2 Department of Global Public Health, Karolinska Institutet, Stockholm, Sweden; 3 Department of Epidemiology and Biostatistics, School of Public Health, Makerere University, Kampala, Uganda; 4 Benadir University Institute of Research & Development, Mogadishu, Somalia; 5 Department of Nutrition, University of Kinshasa, Kinshasa, Congo (the Democratic Republic of the)

**Keywords:** Health policy, Interdisciplinary Research

**Summary box**

- Despite notable progress in some health-related targets, the majority of health-related Sustainable Development Goal (SDG) targets are off track in fragile and conflict-affected countries, which are simultaneously facing geopolitical insecurity and declining funding sources.
- Although there is a clear recognition of the interconnectedness of different SDGs and health, understanding how these linkages play out in practice and how they can be leveraged to accelerate action remains limited in fragile and conflict-affected countries.
- Recent evidence from Uganda, the Democratic Republic of Congo and Somalia highlights that most SDGs are mutually reinforcing, with SDG 16 (peace, justice and strong institutions) playing a particularly significant role in promoting both sustainable development and health in these countries, while trade-offs exist, especially between environmental SDGs and those related to industry and the economy.
- To accelerate progress towards the SDGs and ensure no one is left behind in the journey towards 2030 and beyond, it is crucial to act on these linkages through multisectoral collaboration, informed by evidence-based approaches and community engagement.

## Introduction

 In 2015, all countries in the world agreed on a framework for sustainable development: the 2030 Agenda.^1^ A bold vision of how countries together can achieve sustainable development across three dimensions: environment, economic and social. To make the vision come to life, 17 Sustainable Development Goals (SDGs), 169 targets and 231 unique indicators aligned across the three dimensions were agreed. The SDGs and their targets are directly and indirectly connected. Although these interactions are made explicit in the preamble of the 2030 Agenda, this recognition is not reflected in the formulation of the SDG targets and indicators.^2^ While the health-related SDG targets, such as child mortality, have continued to show impressive reductions, the world is not on track to meet around half of the SDG targets, and one-third have not shown any improvements or even regressed since 2015.^3^ Fragile and conflict-affected countries have lagged behind in the progress towards the SDGs. Indeed, many countries are experiencing polycrisis with conflicts, political instability, climate change and the lingering impact of the COVID-19 pandemic, making progress difficult. Fragile and conflict-affected countries will house an increasing proportion of populations who face several overlapping deprivations across the SDGs outside of the traditional public health sphere, from a lack of proper infrastructure development to climate change pressures.^4^ This is also true for health outcomes, with, for instance, maternal and child mortality being considerably higher in fragile or conflict settings, which also face a larger burden of infectious diseases.^5 6^ If the world is to live up to the 2030 Agenda of not leaving anyone behind, there is an urgent need to focus efforts on promoting sustainable development in these countries. The cuts in funding from major sources and lack of geopolitical stability over the last months have already been felt in these countries and threaten to significantly undermine or even reverse improvements made. In an increasingly complex landscape of policy priorities at the country level and constrained resources, it becomes ever more relevant to understand how progress in one sector (health or non-health sector) might lead to positive impacts in another.

### Perspectives on the linkages between the SDGs and health in fragile and conflict-affected countries

Since the SDGs were adopted in 2015, researchers have developed different models to understand the linkages between the SDGs, from more quantitative modelling methods^7 8^ to document reviews.^9 10^ Providing a methodological middle road, the SDG Synergies approach uses the knowledge and experience of a stakeholder group to assess the interactions between SDG goals or targets, explicitly incorporating the context-specificity of the interactions.^11 12^ From the assessment, a score on a scale from −3 to +3 is usually made through a consensus approach. The scores of the interactions serve as the basis for more advanced quantitative estimations of the cumulative impact of the interactions through network analysis. In this way, the individual scoring and the ripple they make through the network can be assessed, calculated and subsequently illustrated.^11 13 14^ A generic approach to describing interactions will not generate scientific evidence that policymakers can use since the strength, position and nature of the interactions are highly context specific.^15^,^17^ Moreover, compared with quantitative modelling and document reviews, the assessment of interactions in the SDG Synergies approach to a large extent follows similar decision-making processes that take place in policy development and prioritisation. The SDG Synergies approach is particularly useful in fragile and conflict-affected countries as it captures the perspectives of a broad group of stakeholders in a resource-efficient way while providing clear context-specific considerations for policy action.

**Figure 1:**
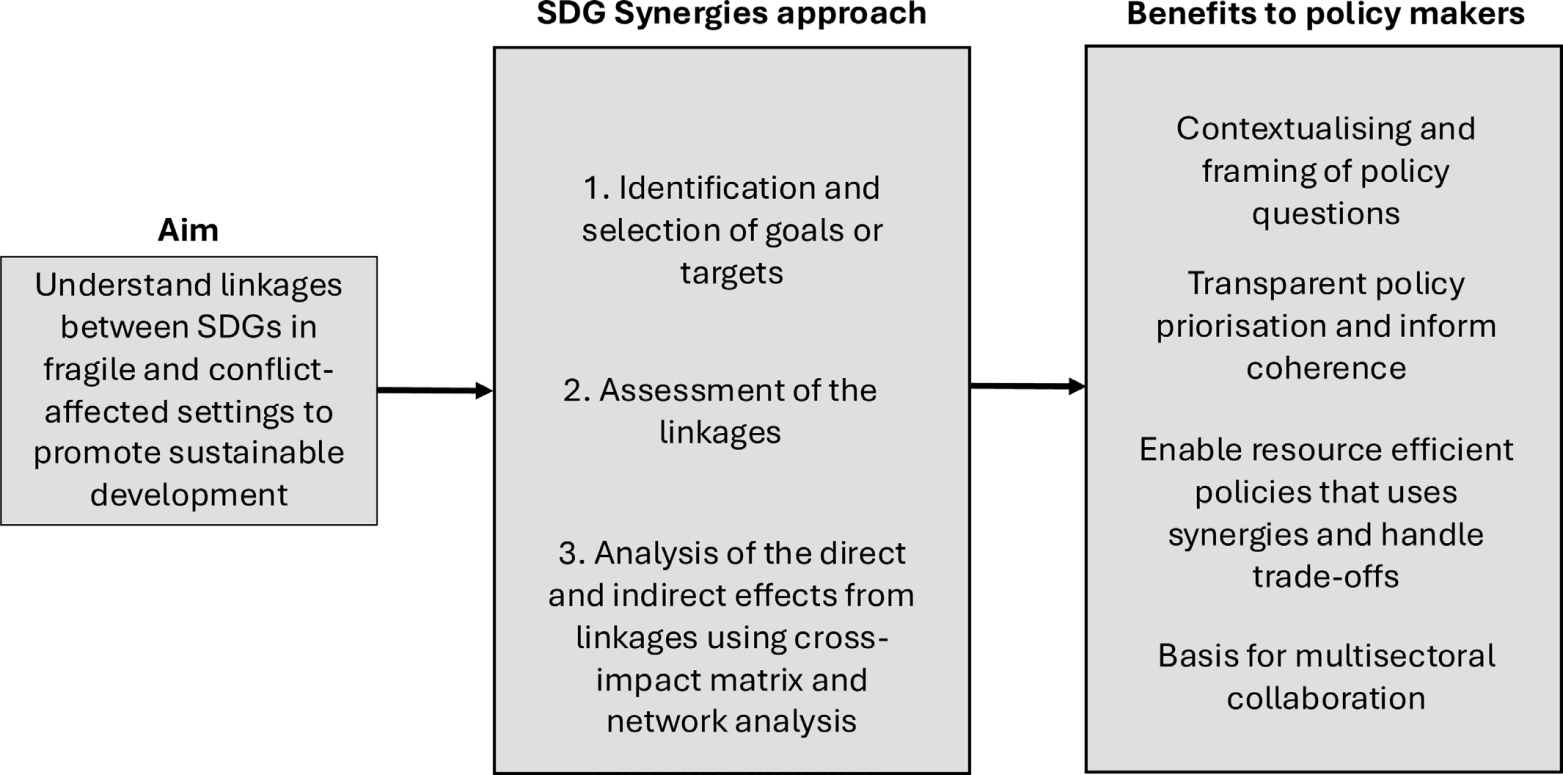
Overview of the activities and possible benefits from applying a Sustainable Development Goal (SDG) Synergies approach.

To inform the understanding of the linkages between health and the SDGs in fragile and conflict-affected countries, we conducted such assessments in Uganda, Somalia and the Democratic Republic of Congo,^18^,^20^ which is synthesised here and when compared provide useful insights. The teams of each country followed the same SDG Synergies approach,^11 21^ that is, a multisectoral group of stakeholders scored 240 unique pairwise linkages between different SDGs, where SDG 3 represented health. Across the three countries, we found a strikingly similar pattern. First, there were mostly positive linkages between the SDGs; the number of negative or restricting linkages was only 2–8%. This strengthens the argument for the overall synergistic reinforcing potential of making progress on all SDGs in fragile settings. SDG 16 (peace, justice and strong institutions) was considered the most important SDG for positively influencing all other SDGs, including health, in Somalia and the Democratic Republic of Congo. The critical role of institutional stability and rule of law for enabling sustainable development is well known,^22 23^ and while not a fast or easy process,^24^ it is key to unwinding the vicious cycle of conflict, poverty and instability in conflict-affected countries.^25^ The different targets of SDG 16 have broad application across every aspect of sustainable development and health, from strong institutions to the reduction in violence and corruption.^26^ There are several challenges facing the successful implementation of SDG 16; however, the importance of SDG 16 underpinning progress on other SDGs cannot be overstated.^27^ In Uganda, a relatively more stable country with less conflict, SDG 16 was deemed the second most important SDG after SDG 10 (reduced inequalities) for overall sustainable development, which might reflect the growing concern of inequality as a hindrance to acceleration within a range of sectors, including health. Moreover, there is ample research on the connections between inequality and risk of eroding institutional stability, with many arguing that one of the key drivers of sustained progress and reduction of risk of conflicts is improved equality.^28^ Across the countries, the negative or restricting linkages found were often those between making progress on the economic or industry-related goals (SDG 8 and 9) and environmental goals (SDG 13, 14 and 15). The tension between economic and environmental priorities has become increasingly evident with accelerated climate change that affects large parts of populations in these countries simultaneously, as poverty and poor economic performance hinder investments. By investing in climate change, sound economic development and ensuring that mitigation and adaptation measures strengthen local economies, there are substantial opportunities for turning this perceived trade-off into impactful cobenefits.^29^ Across the three countries, health was seen as being one of the SDGs being influenced the most by the progress (or lack thereof) of other SDGs. In contrast, SDG 3 was not among the top-rated SDGs to positively influence other SDGs. One possible explanation for this is that the existing state of health and well-being might determine the possibility of advancing SDG 3 in these three countries. While it is undoubtedly important to focus on strengthening health system resilience and healthcare delivery,^30^ improvements in SDG 3-related areas alone are unlikely to drive broader sustainable development objectives and must be complemented by concurrent efforts across multiple goals to drive sustainable gains, including in health outcomes. This underscores the imperative for a comprehensive understanding of SDG interdependencies and highlights the necessity of a synergistic approach to sustainable development that incorporates broader social, economic and environmental determinants of health in fragile and conflict settings.^31^,^33^ An overview of the concept, steps of the SDG Synergies approach and benefits to policymakers are presented in figure 1.

### Policy implications for acting on the linkages to accelerate public health progress

Understanding the interlinkages between different sectors is paramount to effectively promoting public health and well-being. The complexity of challenges in these settings necessitates a comprehensive approach that acknowledges the interconnectedness of various sectors and priorities. In particular, making explicit synergies and trade-offs between different policy areas is key. To achieve sustainable improvements in public health in fragile or conflict-affected contexts, interventions must address the underlying determinants of health, which extend beyond the health sector alone. By embracing a cross-sectoral perspective, policymakers, researchers and other stakeholders can contribute to building resilient communities and fostering long-term development trajectories. Perhaps most importantly, the understanding of the interactions and, correspondingly, how to act on them is primarily at the local level; therefore, locally developed and context-specific solutions are key. For instance, the Somalia Multisectoral Nutrition Strategy was designed taking into consideration the unique interactions driving food insecurity and offered a comprehensive multisectoral response tailored to the local context.^34^

Promoting health and sustainable development requires innovative approaches that transcend traditional sectoral boundaries and the same holds true in fragile and conflict-affected countries. Multisectoral platforms and collaboration offer promising strategies to address complex challenges and foster holistic development agendas. Multisectoral collaborations serve as pivotal mechanisms for integrating diverse perspectives and expertise to address complex issues such as health, education, livelihoods and governance in fragile settings.^35^ These collaborations can facilitate coordination and alignment of efforts across sectors, ensuring a comprehensive response to interconnected challenges. For instance, initiatives that combine health interventions with nutrition programmes can yield synergistic benefits, contributing to improved well-being and resilience among communities affected by conflict.^34^ Multisectoral collaborations encourage collective problem-solving and foster innovation by bringing together stakeholders from government agencies, non-governmental organisations, academia and the private sectors. Moreover, they can promote inclusivity by incorporating community voices into decision-making processes, thus enhancing ownership and sustainability of development interventions. Multisectoral collaboration transcends the limitations of siloed approaches by fostering partnerships and promoting shared responsibility for development outcomes. In fragile and conflict-affected countries, collaboration across sectors is indispensable for addressing the root causes of instability and promoting resilience. However, they are also difficult to implement, and it is essential that technical expertise and effective interventions from single sectors or traditional vertical programmes do not become diluted or omitted. Effective multisectoral collaboration hinges on building trust and mutual understanding among stakeholders, overcoming institutional barriers and aligning incentives.^36 37^ Academia has been shown to be a good entry point to and mediator in multisectoral collaboration since universities in these contexts are often seen as neutral and trustworthy. Coordinated action enables the pooling of resources, expertise and capacities, leading to more sustainable and equitable outcomes. Moreover, collaborative approaches enhance accountability and transparency, essential elements for promoting good governance and reducing corruption in fragile settings.^37 38^

Multisectoral platforms provide opportunities to leverage resources efficiently and maximise impact, which can be utilised in fragile and conflict-affected countries. Understanding how different linkages work together can inform the prioritisation of resources. This understanding could be accompanied by more structured cost-effectiveness analyses, which are vital in informing decision-making processes within multisectoral development initiatives in fragile contexts. These analyses help prioritise interventions based on their potential impact, cost-effectiveness and sustainability.^39^ In conflict-affected countries, where resources are even more scarce than in other settings and competing priorities abound, conducting rigorous cost-benefit or cost-effectiveness evaluations is crucial for optimising resource allocation, and in this perspective, the linkages between different sectors must be incorporated. This approach fosters efficiency and accountability, ensuring that limited resources are allocated to initiatives with the highest potential for positive impact while also showcasing the devastating cost of inaction. Furthermore, understanding the linkages between the SDGs for priority setting will provide valuable insights into the long-term implications of health interventions, guiding strategic planning and adaptive programming in often-changing societal landscapes. Importantly, equitable partnership, reciprocal learning and capacity building led by country stakeholders are crucial in improving health outcomes in conflict and fragile settings.^40 41^ In this endeavour, community engagement and empowerment are integral components of capacity building. By involving local communities in the activities, capacity building initiatives can create sustainable solutions that resonate with the needs and priorities of affected populations.^31^

## Conclusions

To ensure that an acceleration towards improvements in public health can happen and the promise of the 2030 Agenda is upheld, significant action must be taken to sustain and direct increased funding and efforts while, more importantly, moving from understanding to action on health and the SDGs in fragile and conflict-affected countries is needed. Promoting multisectoral collaboration through structured platforms and informed decision-making processes is essential for advancing public health and sustainable development in these settings. Multisectoral approaches enable holistic responses to complex challenges, while collaboration across sectors enhances the efficiency and effectiveness of interventions.

## Data Availability

There are no data in this work.
